# Effect of Food-Simulating Liquids on the Leachability of Plasticizers from Dental Tissue Conditioners

**DOI:** 10.1055/s-0043-1777046

**Published:** 2024-02-08

**Authors:** Wan Nor Syariza Wan Ali, Sandra Parker, Mangala Patel

**Affiliations:** 1 Department of Conservative Dentistry and Prosthodontics. Universiti Sains Islam Malaysia, Kuala Lumpur, Malaysia; 2 Institute of Dentistry, Faculty of Medicine and Dentistry, Queen Mary University of London, London, United Kingdom

**Keywords:** tissue conditioner, plasticizer leaching, food-simulating liquids, Shore A hardness

## Abstract

**Objective** Tissue conditioners are composed of poly(ethyl methacrylate) (PEMA) powder and plasticizer/ethanol mix liquid. Butyl phthalyl butyl glycolate (BPBG) plasticizer is commonly used in tissue conditioners, but the main concern with phthalate plasticizer is its leachability and biocompatibility, especially the estrogenic activity and cytotoxicity of phthalate. Therefore, acetyl tributyl citrate (ATBC) plasticizer has been introduced and formulated as plasticizer in tissue conditioner; however its leachability characteristics are still unknown. Furthermore, the effect of foodsimulating liquids toward leachability of BPBG and ATBC plasticizers has not been documented. The objective of this study was to compare the effect of food-simulating liquids on the leachability of plasticizers and hardness of two experimental tissue conditioners containing BPBG and ATBC plasticizers.

**Materials and Methods** Ten experimental materials were prepared using PEMA polymer powder with 95% plasticizer (BPBG and ATBC) and 5% ethanol by volume, using powder to liquid ratio of 1.67:1, and the thickness was controlled at 3 mm. Shore A hardness value was measured after immersion in distilled water, artificial saliva, 25% ethanol/water mix, 3% citric acid, and coconut oil at 37°C. Measurements were taken at 2 hours and 1, 2, 3, 4, 7, 10, 14, 21, 28, 42, 56, and 84 days. Six readings were taken for each sample and hardness change was calculated and statistically analyzed using Wilcoxon signed-rank test.

**Results** Increase in hardness value was noted for both plasticizers over time with the highest increase was when immersed in coconut oil. Shore A hardness value was significantly higher in ATBC after 84 days of immersion in all food-simulating liquids. The increase in hardness is due to plasticizer/ethanol leaching; however, as ethanol content was the same (5%), the hardness change is attributed to the leaching of plasticizers.

**Conclusion** Leaching of both plasticizers was highest in coconut oil indicating that tissue conditioners may have a shorter intraoral lifetime in patients eating high-fat diet.

## Introduction

Tissue conditioners are soft polymer gels which are commonly used chairside for various applications in dentistry. It can be used to improve the fit of a denture and treat traumatic oral mucosa due to ill-fitting denture by acting as a “cushion” to allow the damaged tissue to return to normal and distribute the occlusal forces. 1 The resilient nature of tissue conditioner will absorb the mechanical stresses during mastication and allow healing of traumatized tissue. 2 The use of tissue conditioners as drug-delivery vehicle such as antifungal 2 and chitosan oligosaccharide 3 is also being explored to treat denture-induced stomatitis.

Tissue conditioners are composed of poly(ethyl methacrylate) (PEMA) powder and plasticizer/ethanol mix liquid, without the presence of monomer. During gelation, plasticizers will lubricate the movement of polymer chains by penetrating and weakening the intermolecular bonding of the polymer chains and dissolving the polymer, producing a non-cross-linked amorphous polymer. 4 In the mouth, when tissue conditioner is in direct contact with the oral fluid, ethanol and plasticizer will leach out and water/fluid will be absorbed, 5 resulting in a less viscoelastic material. The water absorption and plasticizer leaching will impact the susceptibility, flexibility, surface roughness, and stability of the relining materials. 6 Leachability of plasticizers depends on their molecular weight; higher molecular weight leaches less than the lower molecular weight plasticizer, thickness of the tissue conditioner, physical properties of the material, presence of saliva, and repeated loading or mastication. 7

The most commonly used plasticizer is phthalate based, which includes butyl phthalyl butyl glycolyl (BPBG), butyl benzyl phthalate, and butyl benzoate and ester of stearic acid. 8 The main concern with plasticizers in tissue conditioner is its leachability. Leaching of plasticizer will harden the tissue conditioner, resulting in altered physical and mechanical properties of the material.

Another concern with leached plasticizer is its biocompatibility especially phthalate, due to its estrogenic activity and cytotoxicity *in vitro* . 9 According to Nishijima et al (2002), the estrogenicity of a material depends on whether their chemical structures contain benzene rings. 10 Plasticizers without any benzene rings in their structure such as acetyl tributyl citrate, dibutyl sebacate, and tributyl phosphate plasticizers did not show any estrogenic activity compared with plasticizers containing benzene rings such as phthalate esters and bisphenol-A-related diphenylalkanes. 10 Even though there has been no reported incidence of phthalate toxicity in the mouth, the biocompatibility issue of phthalate plasticizers should not be neglected.

Citrate plasticizers offer a more biocompatible alternative to phthalates in tissue conditioner formulations; however, little research had been documented on their leachability from tissue conditioner formulations. Citrate plasticizers are esters of citric acid and consist of linear, aliphatic chains with an even number of carbon atoms. They are usually used in resins for food, pharmaceuticals and medical applications, for example, acetyl tributyl citrate (ATBC), triethyl citrate (TEC), and acetyl triethyl citrate. The development of novel citrate-based dental tissue conditioner is not new, and ATBC has been selected as the plasticizer of choice for the formulations. 11 However, up to present time, the leaching of ATBC plasticizer from tissue conditioner and the effect of diet toward its leachability have not been determined.

Therefore, a study had been conducted to compare the change in hardness in an experimental tissue conditioner containing ATBC plasticizer with tissue conditioner containing BPBG plasticizer over time as well as to compare the effect of food-simulating liquids on the change in hardness of these two tissue conditioners containing BPBG and ATBC plasticizers.

## Materials and Methods

### Material Preparations

Two experimental tissue conditioners were developed for this study using PEMA with 95% plasticizer and 5% ethanol by volume, using BPBG and ATBC plasticizer.

Total 50 mg of PEMA powder (TS1364 polymer powder from Leucite International) was placed in a ceramic container (Pascall Engineering, Sussex, UK), with capacity of 500 mL together with alumina balls of two different diameters: 7 balls with average diameter of 26.1 mm and 20 small balls with average diameter of 18.9 mm. The total weight of balls was 504.2 g. The ceramic container was clamped tightly using metal clamp on the ceramic lid and placed on the rollers of mill machine (GEC Machines Ltd, Newcastle, UK). The powder was ball milled for 16 hours.

Freshly mixed liquid was prepared using 95% by volume BPBG plasticizer and 5% by volume ethanol content for experimental material A (tissue conditioner A [TCA]) and another liquid containing 95% ATBC and 5% ethanol was prepared as experimental material B (tissue conditioner B [TCB]).

### Pilot Study

A preliminary study was conducted to determine the maximum powder to liquid ratio which is clinically acceptable and the optimum thickness of the tissue conditioner, as the hardness value is dependent on the thickness of the material.

#### Powder to Liquid Ratio

Powder to liquid ratio was determined by mixing TCA and TCB to a clinically appropriate consistency using 2 mL of premeasured liquid. Powder was added to the liquid and the remaining powder was reweighed and the powder to liquid ratio was calculated for both TCA and TCB.

From the pilot study, the powder to liquid ratio was calculated, which ranged from 1.46:1 to 1.67:1 for tissue conditioner containing BPBG (TCA), and slightly lower ratio for tissue conditioner containing ATBC (TCB), which were 1.29:1 to 1.34:1. The maximum powder to liquid ratio (1.67:1) was used in the final study.

#### Optimum Thickness

Experimental materials TCA and TCB were prepared based on the powder to liquid ratio of 1.67:1. The mixed materials were placed in a stainless steel window sized 120 × 70 × 1 mm over a flat metal plate and covered with an acetate sheet. Another acetate sheet was used to cover the mixed materials and a second metal plate was placed on the top of the acetate sheet. Hydraulic pressure of ∼100 bar was applied to the assembly using a hydraulic pressure press and the materials were left to gel. After 2 hours, the gelled sheets were removed and cut into six smaller pieces measuring 4 × 3 × 1 mm. Six Shore A hardness readings were taken at six sites on the surface of each sample. H17A Wallace Shore A hardness tester was used to measure the hardness of the material, and the tester was calibrated according to ASTM D2240 prior to hardness tests. Layers of tissue conditioner were added to the first layer, one layer at a time, until the maximum thickness of 6 mm was achieved. The hardness of the samples was measured at 1, 2, 3, 4, and 6 mm. The optimum thickness for use in the final study was calculated from a hardness versus specimen thickness graph.

**Fig. 1:**
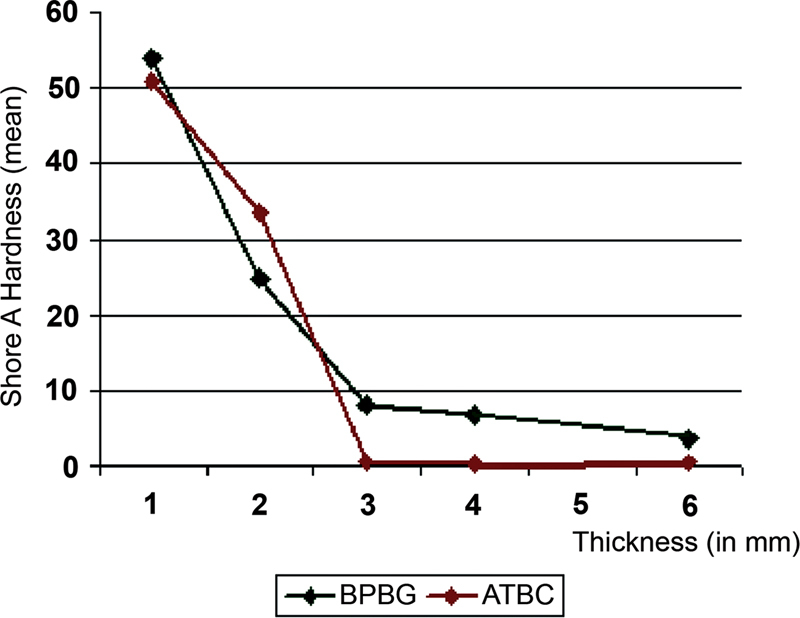
Shore A hardness value in relation to the thickness of the tissue conditioners.

From the test, the Shore A hardness value of both materials decreased as the thickness increased. A great reduction in Shore A hardness value was noted in TCA and TCB when the thickness increased from 1 to 3 mm. However, the hardness was only slightly reduced and leveled off when it was between 3 and 6 mm thick ( Fig. 1 ). Therefore, the thickness of 3 mm was used to prepare the samples for both TCA and TCB in the final study.

### Sample Preparations

**Fig. 2:**
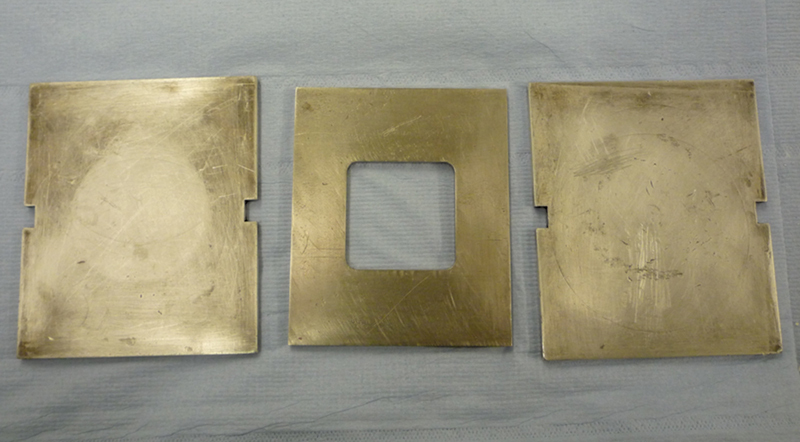
The metal mold used for preparation of samples.

**Fig. 3:**
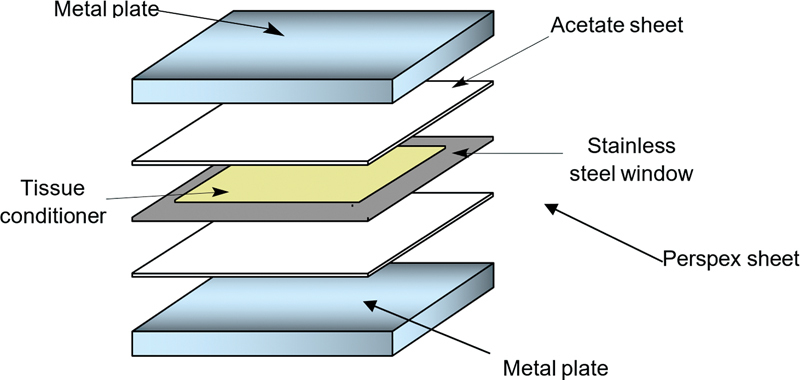
The assembly for sample preparations.

**Fig. 4:**
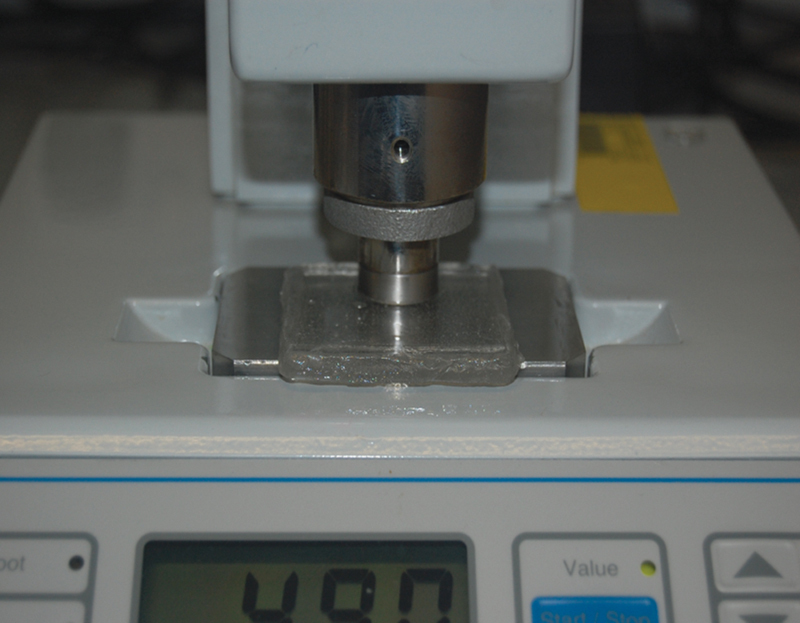
Sample placed on Shore A hardness tester machine.

Ten samples for the two different tissue conditioner formulations (TCA and TCB) were prepared using a powder to liquid ratio of 1.67:1. A mold comprising a metal window sized 50 × 50 × 5 mm was used ( Fig. 2 ) and sandwiched between two metal plates and lined with acetate sheet. Perspex sheet of 2 mm thick was placed in the window and the material was packed on top ( Fig. 3 ), producing samples of 3 mm thick. The assembly was clamped together and put under hydraulic pressure press (Quayle Dental, UK) at ∼100 bar to gel for 2 hours. The final thickness of the tissue conditioner was controlled at 3 mm. The sample was then removed from the mold and the hardness was measured using H17A Wallace Shore A scale hardness durometer tester ( Fig. 4 ) and results were reported in Shore units.

Immediately following the Shore A hardness measurements, each sample was placed in a jar containing 100 mL of one of the immersion liquids with the tissue conditioner placed uppermost and stored at 37 ± 1°C. The food-simulating liquids used were distilled water to simulate aqueous food/control, artificial saliva (Orthana Kemisk Fabrik, Denmark), 3% citric acid (BDH Chemical Co.) to simulate acidic food, 25% ethanol (BDH Chemical Co.) to simulated alcoholic food, and coconut oil (Acros Organic, Thermo Fisher) to simulate fatty food. Two samples of each material were placed in each of the five liquids. Shore A hardness was measured at 2 hours and 1, 2, 3, 4, 7, 10, 14, 21, 28, 42, 56, and 84 days. The mean of six readings was calculated from each sample/food-simulating liquid and statistically calculated using Wilcoxon signed-rank test.

## Results

**Fig. 5:**
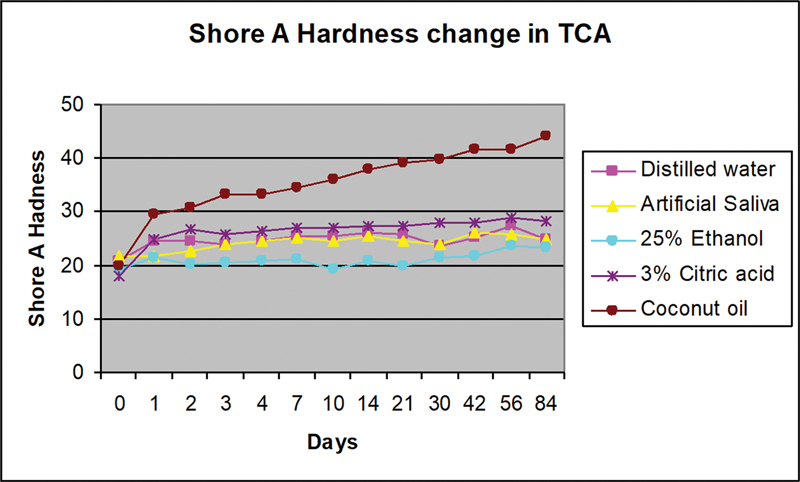
Shore A hardness change of TCA containing BPBG plasticizer after immersion in all food-simulating liquids. BPBG, Butyl phthalyl butyl glycolate; TCA, tissue conditioner A.

**Fig. 6:**
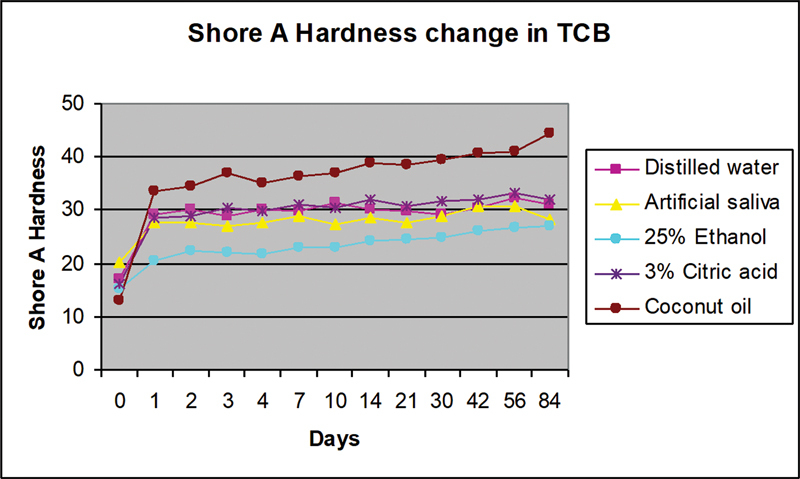
Shore A hardness of TCB containing ATBC plasticizer after immersion in food-simulating liquids. ATBC, acetyl tributyl citrate; TCB, tissue conditioner B.

A total of 10 samples from each formulation were prepared and divided into five different immersion liquids. Twelve readings were obtained from two samples of each tissue conditioner formulation in every immersion liquid. All tissue conditioners in all immersion liquids showed increase in Shore A hardness value after immersion for 84 days ( Figs. 5 and 6 ).

The Shore A hardness value in TCA increased with time for all types of immersion liquids ( Fig. 5 ). The highest increase in hardness value was observed when immersed in coconut oil, with more than 100% increase after 84 days, and the lowest increase was observed in artificial saliva. Immersion in distilled water, artificial saliva, and 25% ethanol showed an increase in hardness of not more than 20%. The percentage of increase in hardness slightly reduced after 56 days of immersion in distilled water, artificial saliva, 25% ethanol/water mix, and 3% citric acid.

After 1 day of immersion, all TCB materials hardened by at least 34% with the least increment in hardness was noted in 25% ethanol and artificial saliva ( Fig. 6 ). The hardness increased for more than 150% 1 day after immersion in coconut oil for 1 day. After 84 days, the highest hardness change occurred after immersion in coconut oil, with an increase of 230% and the lowest increase was in artificial saliva. The changes reduced after 56 days of immersion in distilled water, artificial saliva, and 3% citric acid.

**Fig. 7:**
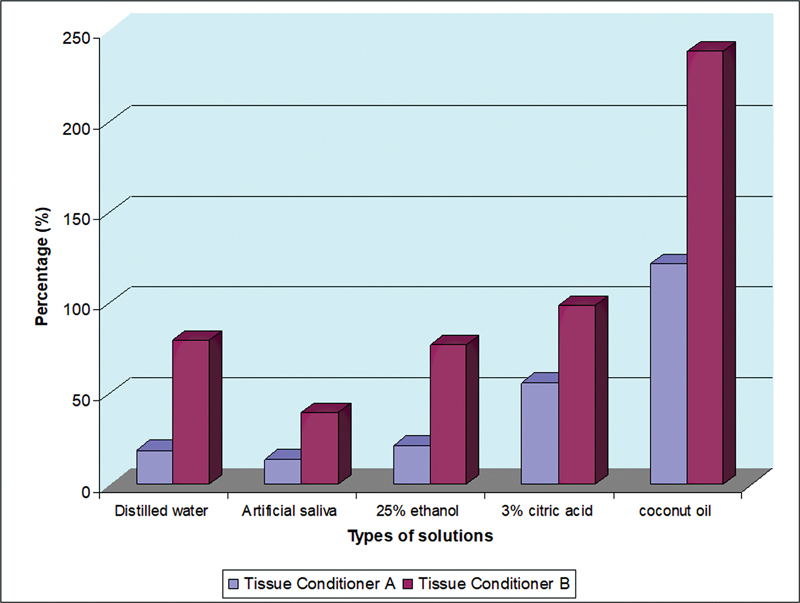
The percentage of changes in Shore A hardness of TCA and TCB after 84 days of immersion food-simulating liquids. TCA, tissue conditioner A; TCB, tissue conditioner B.

After 84 days of immersion in various liquids, the percentage change in hardness for both materials was highest in coconut oil and lowest in artificial saliva ( Table 1 ), and generally, TCB showed more increment in hardness value compared with TCA after immersion in all liquids for 84 days. The highest percentage change was when TCB immersed in coconut oil, with a more than 200% increase in hardness after 84 days. In TCA, the percentage change in hardness was slightly more than 100% after immersion in coconut oil for 84 days ( Fig. 7 ). After 84 days of immersion in distilled water, artificial saliva, and ethanol, TCA showed a change in Shore A hardness value of not more than 50%.

**Table 1:** Differences in Shore A hardness after 84 days immersion in all food-simulating liquids

Immersion solutions	Mean Shore A hardness			
	TCA		TCB	
	At 84 d	Changes from 0 d to 84 d	At 84 d	Changes from 0 d to 84 d
Distilled water	24.92	3.97 (18.95%)	30.92	13.68 (79.40%)
Artificial saliva	24.92	3.05 (13.95%)	28.25	8.07 (39.99%)
25% ethanol/water mix	23.33	4.15 (21.64%)	27.17	11.83 (77.17%)
3% citric acid	28.17	10.13 (56.15%)	32.04	15.96 (98.95%)
Coconut oil	44.17	24.26 (121.85%)	44.5	31.38 (238.99%)

Abbreviations: TCA, tissue conditioner A; TCB, tissue conditioner B.

The Shore A hardness values between TCA and TCB were statistically compared after 84 days in each immersion liquid by using nonparametric Wilcoxon signed-rank test with *p* -value of less than 0.05 was considered as statistically significant.

After 84 days of immersion in solutions, TCB exhibited a significantly higher Shore A hardness when immersed in all immersion food-simulating solutions ( Table 2 ). This indicates that there was a significant influence of the type of tissue conditioner used and immersion fluids used on the Shore A hardness value after 84 days of immersion.

**Table 2:** Shore A hardness value at day 84

	Distilled water		Artificial saliva		25% ethanol		3% citric acid		Coconut oil	
	TCA	TCB	TCA	TCB	TCA	TCB	TCA	TCB	TCA	TCB
Median	18.95	73.48	12.39	37.66	8.19	50.23	49.5	90.58	81.01	182.03
Interquartile range	6.33	6.765	11.39	6.57	9.05	23.94	9.955	16.74	43.72	39.915
Mean difference	−54.61		−27.8		−26.61		−34.15		−84.78	
*p* -Value	<0.05		<0.05		<0.05		<0.05		<0.05	

Abbreviations: TCA, tissue conditioner A; TCB, tissue conditioner B.

## Discussion

The initial compliance of tissue conditioner is determined by the quantity of plasticizers; however, over time, the elasticity reduces due to leaching of plasticizers. 12 In this study, the Shore A hardness values were first measured 2 hours after mixing prior to immersion in food-simulating liquids as baseline value, assuming the gelation process had completed. The completion of the gelation process is important, to ensure the hardness value is not affected by time differences in the stages of the gelation process between the two formulations. According to Parker and Braden (2001), the gelation time reduced with increased milling time of the powder; for example, the gelation of 16 hours milled PEMA powder when mixed with BPBG and 4% ethanol was 5.5 ± 1.0 minutes compared with 13.5 ± 2.0 minutes for the unmilled powder. 8 Therefore, a 16-hour ball-milled polymer powder was used in this study to reduce the gelation time and to ensure that the gelation had completed within the 2-hour period prior to the first hardness test.

In this study, the hardness of two tissue conditioners containing different plasticizers was compared after immersion in food-simulating liquids. It was noted that at the same powder to liquid ratio, tissue conditioner containing BPBG plasticizer exhibited a higher initial Shore A hardness value compared with tissue conditioner with ATBC plasticizer. However, after 1 day of immersion in food-simulating liquids, tissue conditioner with ATBC plasticizer exhibited a significantly higher Shore A hardness value compared with tissue conditioner with BPBG plasticizer, except in 25% ethanol. The significant change after 1 day of immersion in liquids might be due to incomplete gelation or the difference in gelation process/stage between the two materials after 2 hours. ATBC plasticizer has a higher molecular weight (402 g/mol) compared with BPBG (336 g/mol), which may result in longer gelation times for ATBC compared with BPBG, since higher molecular weight plasticizers will produce significantly longer gelation times. 7 Also, the 2-hour hardness values were determined at room temperature, whereas the 1-day hardness value was measured following storage at 37°C, simulating the body temperature. The increase in temperature during storage may have resulted in further chain entanglement, 7 resulting in significant increase in hardness after 1 day.

After 1 day of immersion, all samples exhibited a gradual increase in Shore A hardness with time, as in agreement with Hong et al (2012). 13 The profile of this increase for both materials are similar, except that higher hardness values, was noted in tissue conditioner containing ATBC plasticizer. All samples exhibited an increase in Shore A hardness after 84 days immersion in all liquids as in agreement with Mese and Guzel (2008) who found that the hardness value of soft lining materials (Meliodent, Vertex Soft, Coe-Soft, Molloplast B, and Mollosil Plus) was higher with increased duration of immersion. 14 A study by Khaledi et al (2015) also found significant increase in hardness value of Mollosil soft liner when immersed in food-simulating agent such as heptane, citric acid, and 50% ethanol, resulting in decrease in tensile bond strength. 15 It was also suggested that leaching of plasticizers from aqueous solutions is also osmotically dependent. 15 Our study used the same solutions throughout the study period, and it is acknowledged that the results obtained might be different if the solutions were changed everyday as it may become saturated with plasticizers thus reduce its leachability. However, it was felt that the volume of liquid (100 mL) used was such that it would minimize this effect.

The viscosity and increase in hardness of both materials in all liquids with time can be attributed mainly due to the loss of ethanol and plasticizer from the specimens. 7 In this study, the power to liquid ratio and ethanol content in both formulations were the same; therefore, it can be suggested that the change in hardness is mainly due to loss of plasticizers. Two concurrent processes will occur when tissue conditioners are immersed in water/solutions, which are leaching out of ethanol and plasticizers into the solutions and sorption of water by the polymeric phase of the gel. 6 16 The leaching of ethanol and plasticizers results in shrinkage and hardening of the material while absorption of water will lead to expansion and softening of the material. The shrinkage of the tissue conditioners would only occur if the percentage of solubility was higher than percentage of absorption, and vice versa.

Tissue conditioner with ATBC plasticizer exhibited significantly higher percentage change in Shore A hardness after 84 days of immersion in all liquids, resulting in significantly harder material than tissue conditioner containing BPBG plasticizer after 84 days of immersion in all liquids except coconut oil ( *p* < 0.05). The hardening effect might be due to the difference in the molecular weight as well as molecular structures/shape of the plasticizer. Higher molecular weight plasticizers will produce stronger gel formation, resulting in increased hardness. 7 The molecular weight of BPBG is 336.19 g/mol, compared with ATBC 402.5 g/mol, hence a possible explanation for the difference in hardness value between the two materials. The chemical structure of BPBG consists of benzene ring, making it bulkier than the long chain molecular structure of ATBC. Therefore, ATBC might be more easily leached compared with BPBG despite the higher molecular weight. This was in agreement with Kawaguchi et al (2004) who reported that the leaching behavior of phthalate esters from tissue conditioners depends on the chemical structure and also the solubility of the phthalate esters in the immersion medium. 17

Even though tissue conditioner is considered as a temporary relining material that needs to be changed every 7 to 10 days, but our study collected the data up to 84 days of immersion, to study the pattern of hardness change and leachability of plasticizers when immersed in food-simulating solutions. It was noted that the major changes in hardness occur during the first 7 days, followed by slight increase in hardness change after day 7. This is in agreement with Kawaguchi et al (2004) who found the leachability of plasticizers (BPBG and DBP) from denture liners into water was highest during the first week. 17 Significantly higher Shore A hardness value in tissue conditioner containing ATBC plasticizer might be due to insolubility of BPBG in water compared with ATBC, resulting in higher leachability of ATBC compared with BPBG in distilled water.

Based on preliminary study, the thickness of tissue conditioner is controlled at 3 mm. This thickness is also clinically relevant and concurrent with other studies who suggested for the tissue conditioner to be at 2 to 3 mm thick, to obtain its optimum compliance and best physical properties. 18 Our study used Perspex sheet of 2 mm thickness to replicate the denture bases clinically. The use of Perspex sheet is to ensure that the change in Shore A hardness reading is due to leaching of plasticizer, not from the effect of mechanical change from PMMA denture base material. Even though similar flexural strength was found in heat polymerizing PMMA denture base material, 19 the influence of curing method, degree of polymerization, and storage of the material toward the mechanical properties of PMMA denture base material must be taken into consideration to prevent bias in this study. 20 21 Adequate thickness of prosthesis base is also important as plasticizer leaching from tissue conditioner can also diffuse into the acrylic material resulting in deformation and fracture of the dental bases. 6 12

Distilled water was used in this study to provide information on the effect of tissue conditioners in water, without any chemical influence. Artificial saliva used in this study is a chemical solution that is widely used in xerostomic conditions. Even though the artificial saliva is formulated to mimic saliva, it was not a true representation of the composition of the salivary fluids; therefore, the *in vivo* changes of the tissue conditioners might be slightly different from the findings in this study. Acidic food was represented by the 3% citric acid solution, while coconut oil was to simulate fatty food. Our study used only 25% ethanol/water mixed, since most alcoholic beverages do not exceed 25% of ethanol content, and this is also the mid-range value between wine (11.5–17.5%), beer (4%), and spirit (38%).

Significant change in hardness with the highest percentage change was noted when the specimens were immersed in coconut oil for both materials, suggesting that in a diet with high in fat and alcohol, the physical properties of tissue conditioner might deteriorate rapidly, thus requiring frequent review and replacement of the tissue conditioners. This finding is in agreement with other studies which found that significant change in hardness and compliance of temporary soft lining materials was noted when immersed in fatty food simulation solutions such as corn oil 22 and hexane as substitute for fatty foods. 12 15 Immersion of temporary soft lining materials in hexane as fatty food simulant for 28 days might reduce its compliance by 50%. 23 Findings from our study also suggested that coconut oil can be used as a fatty food simulant to test the effect of indirect food additives that often affect the polymers especially tissue conditioners in the mouth. Due to increase in hardness and loss of elastic property of the tissue conditioner, the useable period of tissue conditioner in the mouth should be relatively short. 24 Therefore, there is a need for a more frequent review and regular replacement of tissue conditioner linings especially in patients consuming high-fat diet.

## Conclusion

The leaching of ethanol and plasticizer and water absorption will affect the long-term dimensional stability of tissue conditioners. The rate of plasticizer leaching depends on the types of plasticizer and diet of a patient. The highest increase in hardness in both types of plasticizer was noted in coconut oil (fatty food), where with rapid changes occurred during the first week followed by gradual increase in hardness over time. Therefore, in order for the tissue conditioner to function at its best in the mouth, it is suggested for frequent review and periodic replacement of tissue conditioners especially for patients with high-fat diet.
